# Clinical and psychosocial variables associated with behavioral intentions to undergo surveillance endoscopy

**DOI:** 10.1186/1471-230X-14-107

**Published:** 2014-06-10

**Authors:** John M Hollier, Marilyn Hinojosa-Lindsey, Shubhada Sansgiry, Hashem B El-Serag, Aanand D Naik

**Affiliations:** 1 Center for Innovations in Quality, Effectiveness and Safety (IQuESt), Michael E. DeBakey Department of Veterans Affairs Medical Center, (152), 2002 Holcombe Blvd, Houston, TX 77030, USA; 2 Department of Pediatrics, Baylor College of Medicine, Houston, TX, USA; 3 Department of Medicine, Baylor College of Medicine, Houston, TX, USA; 4 South Central Mental Illness Research Education and Clinical Center (MIRECC), Houston, TX, USA

**Keywords:** Barrett’s esophagus, Surveillance, Intention, Endoscopy

## Abstract

**Background:**

Many patients with Barrett’s esophagus do not adhere to guideline-recommended endoscopic surveillance. Among patient factors related to cancer prevention behaviors, patients’ stated behavioral intention is a strong predictor of behavior performance. Little is known about the patient factors associated with having a strong behavioral intention to pursue surveillance endoscopy. This study explores the association of clinical and psychosocial variables and behavioral intention to pursue surveillance endoscopy among patients with Barrett’s Esophagus and no or low-grade dysplasia.

**Methods:**

Potential subjects were screened using electronic medical records of a regional Veterans Affairs Medical Center and a pathologically confirmed Barrett’s esophagus registry. Eligible participants were recruited by a mailer or phone call and completed a questionnaire to measure six distinct psychosocial factors, their behavioral intention to undergo surveillance endoscopy, and various demographic and clinical variables. Univariate and multivariate linear regression identified the relation of behavioral intention with each of six psychosocial variables.

**Results:**

One-hundred and one subjects consented and returned surveys. The analytical sample for this study consists of the 94% of surveys with complete responses to the behavior intention items. Three of the six psychosocial domains were statistically significant predictors of intention in both univariate and adjusted univariate analysis (salience/coherence β = 0.59, 95% CI = 0.45-0.76, *P* <0.01; self-efficacy β = 0.30, 95% CI = 0.10-0.51, *P* <0.01; and social influence β = 0.20, 95% CI = 0.08-0.33, *P* <0.01). In a multivariate analysis only salience/coherence (β = 0.65, 95% CI = 0.42-0.88, *P* <0.01) remained statistically significant predictor of intention.

**Conclusion:**

This study established the validity of a scale to measure psychosocial variables associated with behavioral intentions to undergo surveillance endoscopy. Results demonstrate the importance of assessing self-efficacy, social influences, and bottom-line belief in the value of surveillance endoscopy when evaluating a patient’s likelihood of completing surveillance endoscopy.

## Background

Detection of pre-cancerous lesions in breast, cervix, and colorectal tissues has significantly decreased the occurrence of advanced stage cancers at diagnosis and their associated mortality and morbidity in the United States [1-3]. The prevalence of esophageal adenocarcinoma (EA) is increasing in the United States and it carries an overall 5-year survival of 15-25% [4-6]. Barrett’s esophagus (BE), a precancerous lesion of EA, occurs in the distal esophagus and is characterized by the metaplasia of normal squamous epithelium with intestinal epithelium. BE has the potential to undergo dysplasia and evolve into EA [7]. The annual rate of transformation to EA ranges from 0.5-1% in non-dysplastic BE to 5-10% in those with high-grade dysplasia [8-11].

Clinical practice guidelines recommend surveillance of non-dysplastic BE via esophagoduodenoscopy (EGD) every three years and every six months to one year for low-grade dysplasia [8,12,13]. The pattern of BE surveillance in routine care is variable. In a cohort of over 29,000 patients with BE receiving care in a national integrated healthcare system, less than 45% underwent BE surveillance consistent with clinical practice guidelines [14]. Socio-demographic characteristics of patients and facility-level factors did not explain surveillance patterns in this study. Prior studies have highlighted the importance of insurance type and other financial incentives as drivers of surveillance endoscopy [15,16]. Less is known about the psychosocial promoters of BE surveillance behavior beyond perceptions of risk and anxiety [17].

The health promotion and disease prevention literature has established the most predictive determinant of whether that individual performs a specific health behavior is that individual’s behavioral intention (i.e., one’s stated motivation to perform that specific action) [18]. The theory of reasoned action has further elaborated a conceptual model of psychosocial variables that moderate the relationship between one’s behavioral intention and the likelihood of carrying out a particular health behavior [19]. Applications of this theory include studies of colorectal cancer screening, condom use, adolescent sexual behavior, smoking, prostate cancer screening, and HIV treatment adherence [20-27]. For example, a prospective study of colorectal cancer screening behavior found that participants’ behavioral intentions, measured by a self-reported questionnaire, was predictive of subsequent colorectal cancer screening initiation and maintenance [28]. This measure of behavioral intention was correlated with a number of psychosocial and behavioral constructs including perceived susceptibility to colorectal polyps and cancer (perceived susceptibility), belief in the effectiveness of colonoscopy screening to detect colorectal cancer (efficacy of testing), one’s “bottom-line” perception about the value of colorectal cancer screening (salient/coherence), confidence in one’s ability to perform the behavior assuming that one wanted to do so (self-efficacy), and perception about what referent others (e.g., family friends, respected experts, etc.) think and do with regard to performing the behavior (social influence) [21,28].

**Figure 1:**
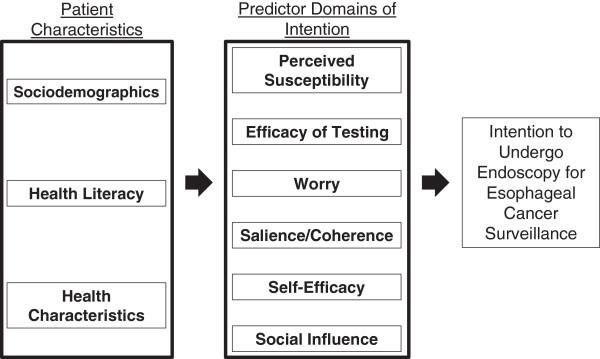
**Conceptual model of factors associated with behavioral intention to undergo endoscopic esophageal surveillance.** This model illustrates the key domains that impact one’s intention to pursue esophageal cancer surveillance. Based on the theory of reasoned action, patient characteristics and psychosocial domains influence the overall behavioral intention to perform a health behavior which is undergoing esophageal cancer endoscopic surveillance in this study.

Building from this evidence base, we propose a conceptual model (see Figure 1) of how clinical, demographic and psychosocial variables relate to patients’ behavioral intention to undergo endoscopy for esophageal cancer surveillance [29,30]. Furthermore, the variables in our model are consistent with survey variables used in the prior study modeling behavioral intentions for colorectal cancer screening [21,28-30]. The aim of the current study is to explore the relationship between these clinical and psychosocial variables and patients’ behavioral intention to undergo surveillance endoscopy. Patients with pathologically confirmed BE received a questionnaire eliciting their intention to undergo endoscopy and responses to the psychosocial variables described in our conceptual model (Figure 1).

## Methods

### Participants and data collection

Potential participants were identified and recruited in two ways. First, we searched electronic medical records (EMR) of a large regional Veterans Administration medical center to identify patients scheduled for EGD for the purpose of BE surveillance. Inclusion criteria for eligible patients included those who were previously diagnosed with BE with low-grade or no dysplasia by histology and actively under surveillance for cancer transformation. Eligible patients received an opt-out letter explaining the study. We contacted patients who did not opt-out and mailed study materials to willing respondents. This method resulted in 68 participants (a 40% response rate for those approached). The second method of recruitment utilized a BE patient registry to identify eligible participants. All of these participants also had a pathologically confirmed diagnosis of BE. Patients were mailed a study information letter and survey. We received a total of 66 surveys from two rounds of mail outs (a 34% response rate for this portion of the sample).

We then conducted a secondary chart review to ensure all participants returning surveys met the BE surveillance criteria. We define BE surveillance as an EGD procedure occurring after a previous EGD resulting in a pathologically confirmed BE diagnosis [29]. Exclusion criteria for participants included the following: (1) under 18 or older than 80 years of age, (2) severe medical or psychiatric co-morbidity, (3) were hospitalized at the time of recruitment, (4) previous BE or gastroesophageal disorders that would require endoscopy for reasons other than BE screening or surveillance (i.e. gastroduodenal cancer, squamous cell esophageal cancer, gastric ulcers, radiation, caustic ingestion, infectious esophagitis, or HIV), (5) unable to undergo endoscopy (i.e. due to gastric or esophageal surgery, resection, fundoplication, or ulcer surgery), or (6) anemia, bleeding, cirrhosis, or metastatic cancer. This secondary chart review resulted in 33 participant surveys being excluded. A total of 101 participants met all inclusion criteria and were consented, but seven had missing values for the intention measure and were subsequently excluded. The remaining 94 participants constitute the analytical sample of the current study. This study was approved by the Institutional Review Board for Baylor College of Medicine and the Michael E. DeBakey Veteran Affairs (VA) Medical Center research committee.

### Instrument development

A validated instrument used to predict colorectal cancer screening was modified for the development of this survey [21,31]. This instrument was modified to measure patient’s intention to participate in an EGD for BE surveillance and includes items measuring the variables described in Figure 1. The scale includes 21 questions forming 6 subscales or domains shown to predict behavioral intention (one outcome). Respondents used a five-point Likert scale to report their level of agreement with each item, responses ranged from (1) not at all to (5) extremely.

After modifying the scale we then field tested the instrument using cognitive interviewing to test the feasibility and face validity of the survey with a sample of patients (n = 6) being seen in an outpatient GI clinic [32]. After completing the survey, a researcher went through each response item-by-item probing for readability and interpretation of each question. Items and survey instructions were modified based on patient feedback. The participants enrolled in the current study received this modified survey. Internal consistency of the 21-item modified study instrument to measure patient’s intention to participate in an EGD for BE surveillance was measured in our study sample using Cronbach’s alpha. The study instrument had an overall Cronbach’s alpha of 0.81 indicating good reliability in our study sample.

#### ***Dependent variable***

*Intention to complete an EGD* was measured as the average between the two intention questions: “I intend to undergo endoscopy” and “I do not intend to go through endoscopy” (reverse-coded). Each item has a 5-point Likert scale from strongly disagree (1) to strongly agree (5). This intention measure is adapted from a validated measure of colonoscopy intention that was found to be highly predictive of receiving colonoscopy for initiation of colorectal screening (odds ratio 2.34, 95% confidence interval 1.44-3.81) and maintenance screening (odds ratio 1.53, 95% confidence interval 1.03-2.63) in a multivariate, prospective study [28]. All study participants included in our final analysis completed the intention to complete EGD measure.

#### ***Predictors of behavioral intention***

Figure 1 illustrates our conceptual model describing the predictors of *behavioral intention* to undergo surveillance endoscopy. The survey items measuring each psychosocial variable in Figure 1 were adapted from the previously described survey of psychosocial predictors of colorectal cancer screening and surveillance colonoscopy among predominately male autoworkers [33]. *Perceived Susceptibility* (3-items) measures the patient’s belief about their chances of developing EA. *Salience and Coherence (4-items)* is the patient’s belief that completing the EGD is seen as important and makes sense to the patient, i.e., the bottom-line importance of BE surveillance. The patient’s belief in the *efficacy of EGD and the curability of EA* was measured by 4-items. *Self-efficacy* (3-items) represents the patient’s confidence in their ability to complete the EGD or the steps necessary for the EGD. A patient’s level of *worry* about the EGD and the possibility of being diagnosed with EA were measured by 3-items. *Social influence* (2-items) measures a patient’s familial influence and support to complete the EGD (Additional file 1). Data is missing from three respondents for the efficacy of EGD measure and from six respondents for the social influence measure.

#### ***Covariates***

*Background variables* were abstracted from patient electronic medical records as well as from survey items. Background factors included demographic characteristics (gender, age, marital status, income, education, and race), degree of BE (non-dysplastic vs. dysplastic), Deyo comorbidity score, and a literacy score. The Deyo comorbidity score is a modification of the Charlson comorbidity summary score. The score has been used to predict mortality within one year of hospitalization. This score is based on points assigned to fifteen specific ICD-9 diagnostic and procedural codes recorded in a patient’s EMR [34]. Health literacy was assessed by having participants respond to the question, “How confident are you in filling out medical forms?” using a 5-point Likert scale (scaled in ascending order of literacy). This single item question has been validated to estimate functional health literacy among English-speaking patient populations with established, criterion measures of health literacy [35]. Education data was missing for three respondents and functional health literacy data was missing for one respondent.

### Data analysis

#### ***Descriptive statistics***

Patient characteristics were reported using sample distribution (Table 1). Mean/standard deviation was calculated for continuous variables, and frequency for categorical variables. Age and literacy were continuous, whereas race (white vs. others), education (some college or greater vs. high school or less), and Deyo score (0, 1, or >1) were categorical variables.

**Table 1:** Demographic and psychosocial characteristics of Barrett’s esophagus patients (n = 94)

**Demographics**	
Age in years, mean ± SD	63.3 ± 6.7
Gender male, n (%)	92 (97.9)
White race, n (%)	84 (89.4)
Some College or College Degree, n (%)	54 (59.3)
Married, n (%)	55 (58.5)
Functional health literacy*, mean ± SD	3.8 ± 1.2
Deyo comorbidity score, n (%)	
Score = 0	48 (51.1)
Score = 1	21 (22.3)
Score >1	25 (26.6)
Predictors of behavioral intention*	Mean ± SD
Perceived susceptibility	2.3 ± 1.1
Efficacy of EGD	3.4 ± 0.9
Worry	2.0 ± 0.9
Salience/Coherence	4.2 ± 0.8
Self-efficacy	3.9 ± 0.8
Social influence	2.9 ± 1.3

*All measured psychological domains and functional health literacy are scored on a one to five point scale.

SD, standard deviation; EGD, esophagoduodenoscopy.

#### ***Regression analysis***

Univariate analysis was conducted using linear regression models to determine the predictor of participants’ reported intention to undergo EGD. Each behavioral and psychosocial variable (perceived susceptibility, efficacy of EGD, worry, salience/coherence, self-efficacy, and social influences) was assessed in a separate univariate model. We then adjusted each univariate model for patient characteristics including age, race, education, functional health literacy, and Deyo score. Lastly, multiple linear regression was conducted including all behavioral and psychosocial variables described under methods and adjusted for patient characteristics. All analyses were conducted using SAS^®^ 9.2.

## Results

The total sample size for the study is 94 participants. The group had a mean age of 63.4 years old and was predominately male (97.8%). The sample was predominately white (89.4%) and college educated (59.3%). The patients’ BE severity was noted as 81 (86%) with no dysplasia and 13 (14%) with low-grade dysplasia. Most individuals were married (58.5%). On average, participants had moderate to good functional health literacy (score 3.8 along 5-point Likert scale). Comorbidity was generally low in this sample as almost three-quarters of all participants had one or less comorbidity. Participants reported a high overall mean score for intention to undergo endoscopy with most scores falling from moderate to very high (mean = 4.5 ± 0.8).

### Summary of measured psychosocial variables

Distributions for behavioral and psychosocial variables are described in the bottom portion of Table 1. Participants reported low to moderate scores (2.0-3.0) for worry, perceived susceptibility, and social influence. They also reported moderate scores (3.0-4.0) for perceived efficacy of endoscopy and self-efficacy to comply with endoscopy recommendations. Participants reported highest scores for salience and coherence of the importance of endoscopy.

### Regression analyses

Univariate analysis indicated salience/coherence (β = 0.59, p < 0.01), self-efficacy (β = 0.39, p < 0.01), and social influence (β = 0.17, p < 0.01) were predictors of behavioral intention to undergo endoscopy in BE patients (Table 2). Even after adjusting for patient characteristics, salience/coherence (β = 0.60, p < 0.01), self-efficacy (β = 0.30, p < 0.01), and social influence (β = 0.20, p < 0.01) remained significant predictors of intention (Table 2). Perceived susceptibility to esophageal cancer, belief in the efficacy of EGD surveillance, and worry about esophageal cancer were not predictors of intention to undergo endoscopy in either of these analyses.

**Table 2:** Predictors of behavioral intention to undergo endoscopy using univariate and adjusted linear regression models

	**Univariate models**			Adjusted, univariate models*		
**Predictors**	**Parameter estimate**	**95% CI**	**P-value**	**Parameter estimate**	**95% CI**	**P-value**
**Perceived susceptibility**	0.01	-0.13 to 0.16	0.89	0.06	-0.08 to 0.20	0.37
**Efficacy of EGD**	0.12	-0.06 to 0.31	0.19	0.11	-0.07 to 0.29	0.23
**Worry**	0.02	-0.16 to 0.21	0.81	0.10	-0.09 to 0.28	0.29
**Salience/Coherence**	0.59	0.45 to 0.74	<0.01	0.60	0.45 to 0.76	<0.01
**Self-efficacy**	0.39	0.19 to 0.60	<0.01	0.30	0.10 to 0.51	<0.01
**Social influence**	0.17	0.05 to 0.30	<0.01	0.20	0.08 to 0.33	<0.01

*Adjusted for Race, Education, Age, Health Literacy, and categorical Deyo scores of 0, 1, and >1.

CI, confidence interval; EGD, esophagoduodenoscopy.

### Multiple regression analyses

Table 3 describes the results of a multivariate analysis that models the association of participants reported intention to undergo endoscopy with all psychosocial variables adjusting for patient characteristics. This model explains 57% of the overall variance in scores of participants’ intentions to undergo endoscopy. The only significant predictor of intention to undergo endoscopy after multivariate adjustment was salience/coherence (β = 0.65, p < 0.01). Self-efficacy and social influence were no longer significant (p = 0.89 and p = 0.95, respectively).

**Table 3:** Multivariate linear regression analysis of predictors of behavioral intention score

**Predictors of intention score**	**Parameter estimate**	**95% CI**	***P *** **value**
**Demographics**			
** Age**	0.01	-0.01 to 0.03	0.41
** Race**	-0.34	-0.82 to 0.16	0.19
** Education**	0.05	-0.21 to 0.34	0.64
** Health literacy**	0.11	-0.19 to 0.46	0.07
Deyo score*			
** Score = 1**	0.14	-0.19 to 0.46	0.41
** Score >1**	0.30	-0.03 to 0.62	0.07
**Psychosocial variables**			
** Perceived susceptibility**	-0.04	-0.20 to 0.12	0.63
** Efficacy of EGD**	-0.03	-0.18 to 0.13	0.71
** Worry**	0.07	-0.12 to 0.25	0.48
** Salience/Coherence**	0.65	0.42 to 0.88	<0.01
** Self-efficacy**	0.02	-0.21 to 0.25	0.89
** Social influence**	<-0.01	-0.13 to 0.13	0.95

*Deyo score of 0 is the referent group.

CI, confidence interval; EGD, esophagoduodenoscopy.

## Discussion

This study assessed the relationship of six psychosocial variables elaborated in our conceptual model (see Figure 1) to BE patients’ intention to pursue endoscopic surveillance. Our analysis revealed three psychosocial variables significantly associated with behavioral intention after adjusting for patient characteristics: salience/coherence (bottom-line importance of BE surveillance), self-efficacy (patient’s confidence in their ability to complete the steps necessary for surveillance), and social influence (patient’s familial influence and support to complete endoscopic surveillance). In a multivariate model that factored all six psychosocial variables and adjusted for patient characteristics, only salience/coherence remained significantly associated with intention to pursue BE surveillance. Worry and perceived cancer risk, the psychosocial variables commonly attributed to cancer surveillance behaviors, were not associated with behavioral intentions in this study.

Our study results build from and are consistent with other studies evaluating patients’ intentions to pursue gastrointestinal cancer screening and surveillance. The survey items used in this study were adapted from a validated survey of psychosocial variables and behavioral intentions to pursue colorectal cancer screening [20,31]. Our study measured similar psychosocial domains specifically related to behavioral intentions for surveillance endoscopy in a sample of BE patients. The survey was feasible to deliver by mail and in-person, had good internal consistency, and performed consistently with our proposed conceptual model (Figure 1). This survey could be used in future studies exploring patients’ psychosocial and behavioral determinants of endoscopy for BE and cancer surveillance.

Despite these strengths, the current study has limitations. Our cross-sectional design and sampling strategy limited our ability to evaluate a prospective relationship between behavioral intention and receipt of endoscopic surveillance. However, there is a well-established literature in disease prevention, broadly, and cancer screening, in particular, delineating this predictive relationship [18]. Our study findings relied primarily on patient-reported psychosocial variables, which may explain why participants’ responses to our behavioral intention measure skewed towards the higher end of the Likert scale. Despite this limitation, these survey measures were previously validated in a study of colorectal cancer screening and our analytical model did include objective measures of clinical and demographic characteristics.

The study population was composed primarily of predominately white, college-educated, male veterans recruited from a population with a low response rate for study enrollment. Selection bias might be present although the demographics of those recruited were similar to those of individuals diagnosed with BE in the VA national database. Despite this difficulty with recruitment, the enrolled sample mirrors a larger sample of VA BE patients with poor adherence to endoscopic surveillance in regard to demographics including race and gender (83.2% white and 97% male) [14]. The study results may not be generalizable to the US general population in regard to inherent patient characteristics including higher level of education and health literacy. These same variables could impact the relationship of salience/coherence and esophageal cancer endoscopic surveillance when compared to a diverse sample. Additionally, this study only measures psychosocial factors outlined in the theory of reasoned action, but other unmeasured psychosocial domains could influence behavioral intention in this context. Lastly, the findings observed do provide insight about psychosocial factors that predict esophageal endoscopic surveillance in this sample, but the results may not be applicable to other cancer screening or surveillance behaviors.Future studies will need to expand and validate our survey instrument to confirm the relationships in our conceptual model (Figure 1) and the predictive relation of behavioral intention and receipt of surveillance endoscopy. This study established the feasibility of measuring important psychosocial domains that predict intention to undergo BE surveillance. Conversely, we now need to better understand the reasons why patients are non-adherent to guideline-recommended endoscopic surveillance.

## Conclusions

Finally, the results of the current study suggest that physicians should focus less on cancer-related worry and risk when discussing BE surveillance and more on the positive emotions related to confidence to complete endoscopy, bottom-line importance of BE surveillance, and the encouragement of patients’ family members to complete surveillance endoscopy.

## Abbreviations

BE: Barrett’s esophagus; EA: Esophageal adenocarcinoma; EGD: Esphagoduodenoscopy; EMR: Electronic medical record; VA: Veterans affairs.

## Competing interests

The authors declare that they have no competing interests.

## Authors’ contributions

JMH participated in the study analysis and drafted the manuscript. MHL was involved in study design, recruitment, data collection, and data analysis. SS participated in study design and statistical analysis. HEB and ADN jointly conceived the study, contributed to data collection and analysis, and supervised the study design and coordination. All authors read and approved the final manuscript.

## Pre-publication history

The pre-publication history for this paper can be accessed here:

http://www.biomedcentral.com/1471-230X/14/107/prepub

## Supplementary Material

Additional file 1 Items Comprising the Intention to Undergo Esophageal Endoscopic Surveillance in Barrett’s Esophagus Survey, stratified by domain. Click here for file
