# Supplementary material for: Clinical and psychosocial variables associated with behavioral intentions to undergo surveillance endoscopy
Source: BMC Gastroenterol. 2014 Jun 10;14:107. doi: 10.1186/1471-230X-14-107 (PMC4079921; doi:10.1186/1471-230X-14-107)
Supplement: Additional file 1 — Items Comprising the Intention to Undergo Esophageal Endoscopic Surveillance in Barrett’s Esophagus Survey, stratified by domain. [file 1471-230X-14-107-S1.docx]

| **Additional file 1.** Items Comprising the Intention to Undergo Esophageal Endoscopic Surveillance in Barrett’s Esophagus Survey, stratified by domain | |
| --- | --- |
| **Domains** | **Items** |
|  |  |
| Dependent Variable | |
| *Intention* | 1. I intend to undergo endoscopy. |
|  | 1. I do not intend to undergo endoscopy. |
|  |  |
| Independent Variables | |
| *Perceived Susceptibility* | 1. I believe the chance I might develop esophageal cancer is high. |
|  | 1. I think it is very likely that I will develop esophageal cancer. |
|  | 1. I believe that the chance that I will develop abnormal esophageal cells is high |
|  |  |
| *Salience and Coherence* | 1. Having an endoscopy makes sense to me. |
|  | 1. I think the benefits of endoscopy outweigh any difficulty I might have in going through the test. |
|  | 1. Going through an endoscopy is an important thing for me to do. |
|  |  |
| *Efficacy of EGD* | 1. I believe that if I had normal endoscopy results, I wouldn’t have to worry about developing esophageal cancer. |
|  | 1. I believe that endoscopy can help to protect my health. |
|  | 1. I think that when abnormal cells are found, esophageal cancer can be prevented. |
|  | 1. I believe that when esophageal cancer is found early, it can be cured. |
|  |  |
| *Self-Efficacy* | 1. Arranging my schedule to go through an endoscopy is an easy thing to do. |
|  | 1. Finding time to go through endoscopy would be difficult for me to do. |
|  | 1. Going through endoscopy would be difficult for me to do. |
|  | 1. I think that going through endoscopy would be an easy thing for me to do. |
|  |  |
| *Worry* | 1. I am afraid of having an abnormal endoscopy result. |
|  | 1. I am worried that endoscopy will show that I have esophageal cancer. |
|  | 1. I am bothered by the possibility that endoscopy might be physically uncomfortable. |
|  |  |
| *Social Influence* | 1. I want to do what members of my immediate family think I should do about endoscopy. |
|  | 1. Members of my immediate family think that I should go through endoscopy. |
| All items used a 5-point Likert scale | |
